# Generalized Peritonitis Secondary to Spontaneous Rupture of the Urinary Bladder

**DOI:** 10.7759/cureus.60053

**Published:** 2024-05-10

**Authors:** Mahmoud Dabbagh, Amine Maazouz, Mountassir Moujahid, Ahmed Bounaim, Sidi Mohammed Bouchentouf

**Affiliations:** 1 Department of Surgery, Mohammed V Military Training Hospital, Rabat, MAR

**Keywords:** exploratory laparotomy, catheter, urinary tract infection, generalized peritonitis, radiotherapy, spontaneous urinary bladder rupture

## Abstract

A spontaneous rupture of the urinary bladder (SRUB) is an exceedingly rare surgical emergency that might be misdiagnosed, resulting in a high mortality risk. Clinicians should be mindful that secondary peritonitis can occur as a result of a ruptured urinary bladder, which is frequently misdiagnosed and undertreated. The majority of cases are identified during laparotomy.

We report a case of a 70-year-old woman who had irradiation for endometrial cancer 25 years ago and had a history of hypertension, diabetes, and recurring urinary tract infections. The current study sought to determine the etiology of SRUB as well as clinical aspects and diagnostic strategies. She was diagnosed with generalized peritonitis.

An exploratory laparotomy discovered a perforated urinary bladder. Following further care, the patient was released with no further complaints.

## Introduction

The spontaneous rupture of the urinary bladder (SRUB) is an uncommon and possibly lethal condition, accounting for less than 1% of all bladder injuries [1]. It is frequently related to trauma, irradiation, persistent bladder infection, bladder tumor, radiation, or neurogenic bladder, and can sometimes be idiopathic [1].

Despite the use of computed tomography (CT) imaging with contrast, it is commonly wrongly diagnosed, resulting in a high fatality rate.

The diagnosis is complicated and is often made via a laparotomy. It is frequently surgically repaired. However, studies have shown that conservative therapy with indwelling catheterization might also provide satisfactory outcomes in certain instances [2].

We present a rare case of generalized peritonitis caused by SBUR, which was difficult to diagnose as the source of persistent abdominal discomfort. In addition, we provide details to demonstrate the clinical characteristics, diagnosis, and therapy of SRUB.

## Case presentation

A 70-year-old woman with a history of hypertension, diabetes, and dyslipidemia under medical treatment had undergone a hysterectomy and postoperative irradiation for endometrial cancer 25 years ago. Moreover, the patient has reported many episodes of hematuria dating back seven years, associated with recurrent urinary tract infections and urinary incontinence.

She was taken to the emergency department for generalized abdominal pain. The patient denied any history of trauma. Upon arrival at our hospital, she looked ill and pale, febrile with a temperature of 38.7°C, blood pressure of 15/9 mmHg, tachycardia with a pulse of 130/min, respiration rate of 28/min, and ketonuria (3+) with a glucose level of 7 mmol/L (127 mg/dl), which revealed diabetic ketoacidosis (DKA). Her abdomen was soft and flat, with the presence of a diffuse rebound tenderness on physical examination, primarily in the suprapubic region.

Laboratory tests revealed a white blood cell count (WBC) of 14300/ul, serum creatinine at 19 mg/l, urea at 1.22 g/l, hemoglobin at 10.9, random blood glucose at 2.96 g/l, and C-reactive protein (CRP) at 460 mg/l (Table 1).

**Table 1:** Laboratory findings of the patient on arrival.

Parameter	Results	Reference range
White blood cell count (10^3^/uL)	14.3	4.5 to 11.0
Creatinine (mg/L)	19	6 to 13
Urea (g/L)	1.22	0.15 to 0.38
Hemoglobin (g/dL)	10.9	13 to 17
Random blood glucose (g/L)	2.96	0.70 to 1.4
C-reactive protein (mg/L)	460	<5

**Figure 1:**
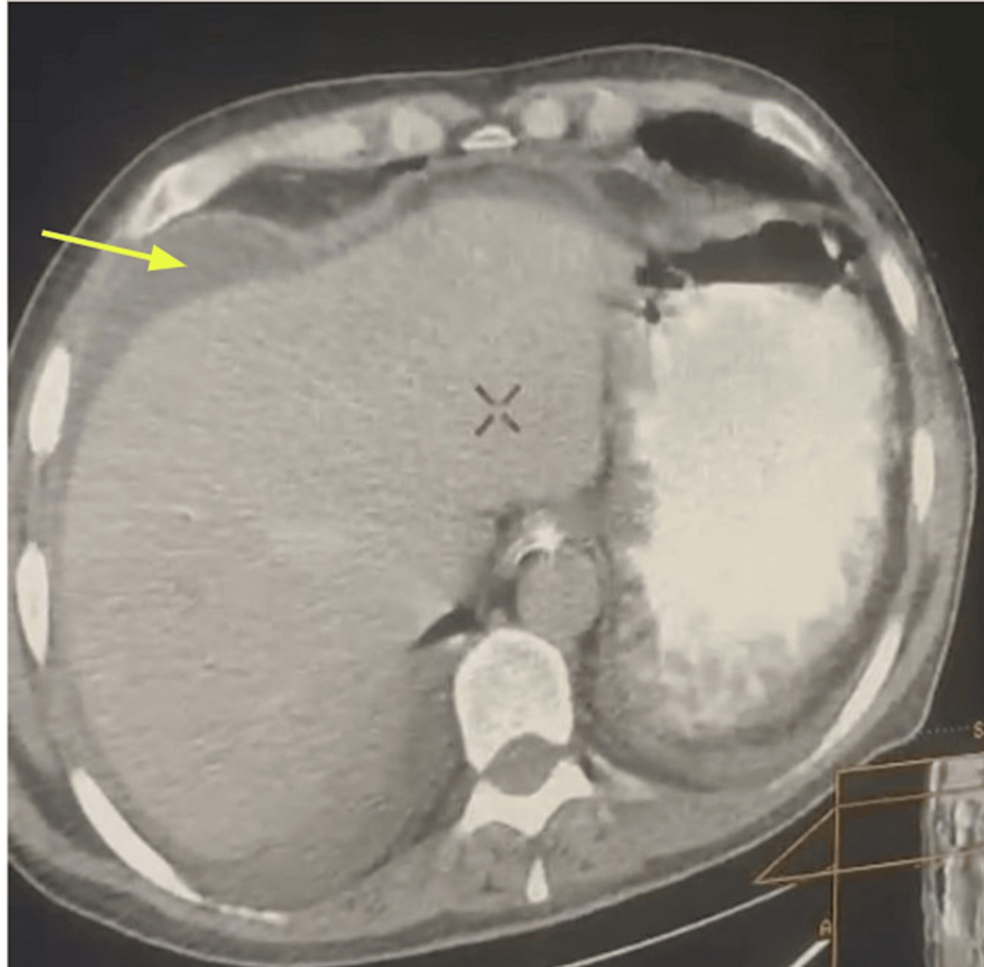
CT scan image revealed an intra-abdominal free fluid (yellow arrow).

An abdominal pelvis CT scan revealed mesenteric fat infiltration with moderate abdominopelvic free fluid but no pneumoperitoneum or hydroaeric levels (Figure 1).

Initially, piperacillin, tazobactam, and amikacin were used for intravenous (IV) rehydration and insulin therapy.

Exploratory laparotomy revealed moderate intra-abdominal seropurulent free fluid, multiple adhesions between the bladder and overlying peritoneum, and a dilated congested bowel loop with no signs of perforation.

**Figure 2:**
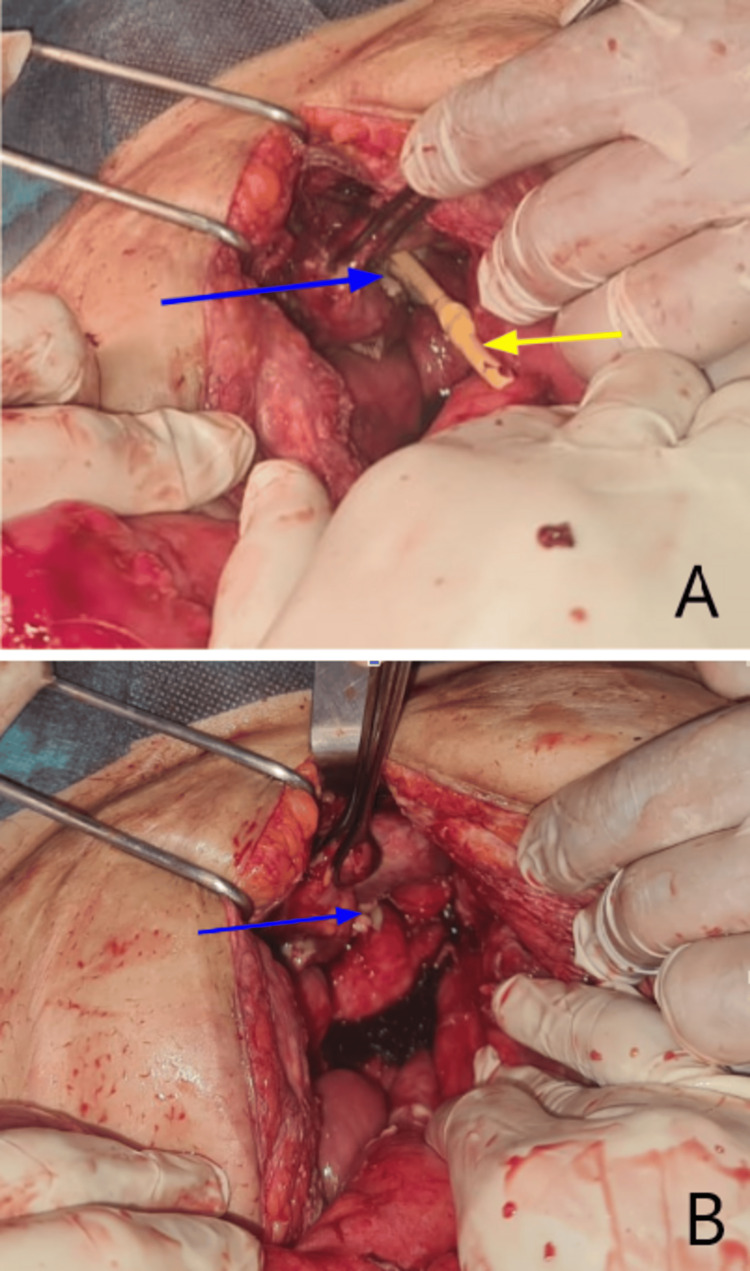
Operative photos showing the bladder rupture (blue arrow in images A and B ), and the Foley catheter balloon floating in the peritoneal cavity (yellow arrow in image A).

A posterior urinary bladder perforation measuring nearly 7.5 cm by 4 cm was discovered, with an inflamed and thickened wall and large defects (Figure 2). Urine was leaking, and the Foley catheter balloon was floating freely in the peritoneal cavity (Figure 2A).

Urologists were summoned for assistance. The rupture repair was deemed impossible due to significant substance losses, the friability of the perforation edges, and its location. We decided to place a J stent on the right ureter because the left side was too difficult to access.

Before a large drainage, which involves two Redon drains placed into the bladder, the peritoneal cavity is thoroughly lavaged. The next day, a left nephrostomy was performed.

The patient's postoperative recovery was uneventful, with obvious clinical and biological improvement. He was admitted to our general surgery department for three days before being moved to the urology ward.

She was discharged from the hospital on the 20th postoperative day and has not had a recurrence since. Three months later, she was scheduled for a cystoplasty.

## Discussion

SBUR is a rare cause of secondary peritonitis. The incidence is approximately one in every 126,000 people [3]. Accurate diagnosis and treatment are critical; however, SBUR is a recurring condition with a poor prognosis if left undiagnosed and untreated. Historically, the mortality rate of spontaneous bladder rupture ranged between 25% and 56% [4], but this has most likely decreased due to improved imaging and management of sepsis and electrolyte imbalances [5]. A missed diagnosis, which causes a delay in treatment, significantly increases mortality. It most commonly occurs at the dome or posterior wall of the bladder.

The causes are classified as traumatic or non-traumatic. Traumatic rupture is the cause of approximately 96.6% of all cases [6]. The majority of spontaneous bladder ruptures are not idiopathic and are caused by tumors, diabetes, radiotherapy, transvaginal delivery, or neurogenic bladder [7]. As a result, making a diagnosis can be challenging. Diabetics with decreased bladder sensitivity, resulting in chronic urine retention and recurred urinary tract infections, may raise the risk of bladder rupture. Radiotherapy has been associated with a spontaneous urinary bladder rupture, and Altman and Horsburgh reported one case in 1966 [8].

Furthermore, a neurogenic bladder can cause spontaneous urinary bladder rupture. Wilson reported a case of neurogenic bladder in 1940 [9]. Approximately 2.1% of patients have bladder injuries after radiotherapy for gynecologic malignancies, and in Japan, SBUR is most commonly observed after pelvic radiotherapy [10]. Our patient is diabetic, and the perforation occurred 25 years after radiotherapy, as previously reported. Infections can occur asymptomatically due to the loss of bladder sensation after hysterectomy, as was the case with our patient, who had recurrent urinary tract infections. SBUR is difficult to diagnose, resulting in delayed treatment [11].

Pelvic radiotherapy can cause bladder complications, including inflammatory infiltrates, fibrosis, necrosis, and cellular atypia.

Urinary bladder rupture generates abrupt abdominal discomfort, which frequently results in the peritoneal irritation sign, rendering it challenging to differentiate it from gastric perforation or other gastrointestinal illnesses. This is why any mistake is critical because delaying diagnosis increases the difficulties.

The most common CT image finding is an accumulation of ascitic fluid. Free air, which strongly suggests a perforated gastric tract, makes preoperative diagnosis difficult. After the laparotomy, a definitive diagnosis was made. Laboratory tests revealed urea (blood urea nitrogen) and creatinine elevations (referred to as pseudo-renal failure). This change is caused by the reabsorption of urine that has seeped into the intra-abdominal space via the peritoneum, although other acute abdomen instances may be accompanied by renal failure, making diagnosis challenging. Consequently, the diagnosis will be delayed and missed by several days [12]. SRUB has a better prognosis when diagnosed and treated early on.

Whatever the etiology, there is usually a root cause that affects the bladder wall and facilitates perforation.

Exploratory laparotomy is the gold standard of diagnosis, as it was used to diagnose practically all documented instances of acute peritonitis. There are no set treatment recommendations for SRUB. According to the European Association of Urology, an intraperitoneal bladder rupture ought to always be treated with routine surgery to fix it since this may result in an illness that could be fatal due to the danger of abdominal sepsis and peritonitis. Alternatively, extraperitoneal bladder rupture is managed conservatively [13]. According to the American Urological Association recommendations, simple extraperitoneal bladder injuries can be treated conservatively with a catheter for many weeks [14]. However, after reviewing the literature, we determined that conservative therapy was an effective treatment for both intraperitoneal and extraperitoneal bladder rupture in select cases that did not include serious infection, bleeding, or substantial damage. Antibiotic medication and proper urine outflow are examples of conservative treatment strategies. Urine can be drained using either a urinary tract catheter or a puncture drainage catheter. Patients with an evident intraperitoneal rupture and no serious abdominal infection or other organ harm can undergo laparoscopic surgery.

An accurate diagnosis, followed by surgical intervention, is the key to a successful outcome.

Given the extent of the bladder perforation in our case, the repair was impossible. Sharing our experience may aid emergency physicians in the early diagnosis and treatment of bladder rupture, thereby avoiding potentially fatal complications.

## Conclusions

Spontaneous urinary bladder rupture is an uncommon emergency that can result in severe abdominal discomfort and septic shock, making it difficult to distinguish from gastrointestinal perforation. It should be suspected in patients with an acute abdomen and a history of recurrent urinary tract infections, pelvic irradiation, or diabetes. CT scan cystography is widely recognized as the most reliable non-invasive diagnostic and assessment method for suspected bladder rupture. Conservative therapy is indicated for those who do not have serious infections, bleeding, or significant damage. Early identification and treatment of SRUB is essential for a smooth recovery. A biopsy of the perforated margins should be performed to rule out cancer.
